# A randomised controlled trial of the clinical and cost-effectiveness of a contingency management intervention compared to treatment as usual for reduction of cannabis use and of relapse in early psychosis (CIRCLE): a study protocol for a randomised controlled trial

**DOI:** 10.1186/s13063-016-1620-x

**Published:** 2016-10-22

**Authors:** Sonia Johnson, Luke Sheridan Rains, Steven Marwaha, John Strang, Thomas Craig, Tim Weaver, Paul McCrone, Michael King, David Fowler, Stephen Pilling, Louise Marston, Rumana Z. Omar, Meghan Craig, Mark Hinton

**Affiliations:** 1 Division of Psychiatry, University College London, London, UK; 2 Mental Health and Wellbeing, Warwick Medical School, University of Warwick, Coventry, UK; 3 Addictions Department, Institute of Psychiatry, Psychology, and Neuroscience, King’s College London, London, UK; 4 Health Service and Population Research Department, Institute of Psychiatry, Psychology and Neuroscience, King’s College London, London, UK; 5 Mental Health Social Work & Interprofessional Learning,, Middlesex University London, London, UK; 6 Department of Health Services and Population Research, King’s Health Economics, Institute of Psychiatry, Psychology & Neuroscience, King’s College London , London, UK; 7 Department of Psychology, University of Sussex, Brighton, UK; 8 Clinical Psychology and Clinical Effectiveness, University College London, London, UK; 9 Department of Primary Care and Population Health and Priment Clinical Trials Unit, University College London, London, UK; 10 Department of Statistical Science, University College London, London, UK

**Keywords:** Financial incentives, Contingency management, Cannabis, Psychosis, Early intervention, Substance misuse

## Abstract

**Background:**

Around 35–45 % of people in contact with services for a first episode of psychosis are using cannabis. Cannabis use is associated with delays in remission, poorer clinical outcomes, significant increases in the risk of relapse, and lower engagement in work or education. While there is a clear need for effective interventions, so far only very limited benefits have been achieved from psychological interventions. Contingency management (CM) is a behavioural intervention in which specified desired behavioural change is reinforced through financial rewards. CM is now recognised to have a substantial evidence base in some contexts and its adoption in the UK is advocated by the National Institute for Health and Care Excellence (NICE) guidance as a treatment for substance or alcohol misuse. However, there is currently little published data testing its effectiveness for reducing cannabis use in early psychosis.

**Methods:**

CIRCLE is a two-arm, rater-blinded randomised controlled trial (RCT) investigating the clinical and cost-effectiveness of a CM intervention for reducing cannabis use among young people receiving treatment from UK Early Intervention in Psychosis (EIP) services. EIP service users (*n* = 544) with a recent history of cannabis use will be recruited. The experimental group will receive 12 once-weekly CM sessions, and a voucher reward if urinalysis shows that they have not used cannabis in the previous week. Both the experimental and the control groups will be offered an Optimised Treatment as Usual (OTAU) psychoeducational package targeting cannabis use. Assessment interviews will be performed at consent, at 3 months, and at 18 months. The primary outcome is time to relapse, defined as admission to an acute mental health service. Secondary outcomes include proportion of cannabis-free urine samples during the intervention period, severity of positive psychotic symptoms, quality-adjusted life years, and engagement in work or education.

**Discussion:**

CIRCLE is a RCT of CM for cannabis use in young people with a recent history of psychosis (EIP service users) and recent cannabis use. It is designed to investigate whether the intervention is a clinically and cost-effective treatment for cannabis use. It is intended to inform future treatment delivery, particularly in EIP settings.

**Trial registration:**

ISRCTN33576045: doi 10.1186/ISRCTN33576045, registered on 28 November 2011.

## Background

Cannabis is the most commonly used drug among people with psychosis, with rates of current use around the time of the onset of psychosis regularly recorded as between 35 and 45 %, well above use patterns in same-age, nonpsychotic populations [1, 2]. Continued use following the onset of psychosis is associated with poorer individual outcomes and greater societal burdens. Hazards include delays in remission, suicidal behaviour, violence and homelessness [1, 3, 4]. In prospective investigations in first-episode psychosis, cannabis use is associated with markedly higher relapse rates: an Australian study reported a 51 % relapse rate over 15-month follow-up among substance users (mostly cannabis) compared with 17 % among nonusers [5], accompanied by a threefold difference in inpatient admission rates. Similarly, a Dutch study reported a 42 % relapse rate among persistent cannabis users compared with 17 % among those who never used or stopped round the time of first onset [3]. A dose-response relationship between severity of cannabis misuse and time to relapse was also reported in this study. Studies of comorbid substance misuse among people with established psychosis indicate that people who persist in problematic drug use are heavy users of acute mental health services, are more likely than others with psychotic illnesses to engage in acts of violence, and are less likely to work, sometimes using disability benefits to sustain drug use [6–8]. Thus, if a reduction in cannabis use can be achieved very early in the course of a psychotic illness, this has potential to improve the life experiences and social recovery of young people who develop psychosis, and to reduce the burden on carers, on mental health, criminal justice and welfare services and on the wider society over many years. This is the overall aim of the current study.

Systematic reviews find that the evidence on effective interventions for comorbid substance misuse in established psychosis is very limited [9, 10]. Despite a promising pilot study [11], a large MRC-funded trial, the MIDAS study, has shown no effect on primary or secondary outcomes from a relatively lengthy intervention (29 sessions over 9 months) consisting of motivational interviewing (MI) and cognitive behavioural therapy (CBT). The difficulties in intervening effectively in established psychosis suggest that it may be fruitful to target an earlier stage of illness, when several recent studies indicate that patterns of use are in a state of substantial flux [12, 13]. Many people are ambivalent about persisting with use and have substantial motivation for change, though some who initially abstain soon return to use [14]. This contrasts with the very limited motivation for change found in established psychosis [15], so that early psychosis may well be a stage at which achieving change with a relatively brief intervention is more feasible. However, in a similar study [16] to the MIDAS trial, a MI and CBT intervention was trialled for cannabis in Early Intervention In Psychosis (EIP) service users, also over 9 months (24 sessions), and again found no benefit for the intervention compared to treatment as usual.

The very limited benefits achieved from psychological interventions, such as MI and CBT in comorbid substance misuse in psychosis, have made us look elsewhere for a potentially effective intervention. Contingency management (CM) is an approach that involves offering rewards contingent on engagement in substance use treatment and on evidence of abstinence. CM is now recognised to have a strong evidence base supporting the efficacy of the intervention in a range of contexts, such as smoking cessation [17] and substance misuse disorders [18], and its adoption in the UK is advocated in guidance issued by the National Institute for Health and Care Excellence (NICE) [19]. However, with the exception of a small number of recently evaluative studies in Europe [20], the evidence base is drawn almost entirely from the US. There is relatively little UK experience of using CM, with only a few evaluations of CM reported. Recent examples include the CONMAN trial, which provided an evidence base for CM in the uptake of hepatitis B vaccines among opiate users [21], and FIAT, which found incentives to be effective for reinforcing adherence to antipsychotic medication [22]. The NICE review of psychosocial interventions [19] identified 14 trials of CM, all from the US, that met criteria for inclusion, of which three involved cannabis use. A consistent finding of a benefit for CM was reported, with most studies using abstinence at 12 weeks as their outcome measure.

Just one North American CM study has so far been reported among people with comorbid substance misuse and psychosis; the substances included were cocaine, heroin, and cannabis. This was unusual among treatment studies in this population in finding a positive effect. Bellack et al. [23] reported that CM, combined with a psychological intervention, resulted in more drug-free urine samples than an enhanced treatment as usual intervention (Supportive Treatment for Addiction Recovery), and in reduced hospitalisation, and a better quality of life. However, only a small proportion of participants abused cannabis (7 %) with 93 % abusing cocaine or heroin. Sigmon et al. [24, 25] performed two small feasibility studies using a within-subjects reversal design that also reported a beneficial effect from the intervention. We find no other evidence of CM studies for cannabis use in a population with psychosis.

In the present study, we will investigate the clinical and cost-effectiveness of CM for reducing cannabis use among EIP service users. This will be evaluated in terms of clinical service use, the presence of psychotic symptoms, cannabis use, and health economic measures. The primary outcome will be whether CM improves time to relapse, measured as admission to acute mental health services, compared to recommended standard care.

## Methods/design

### Design

**Fig. 1:**
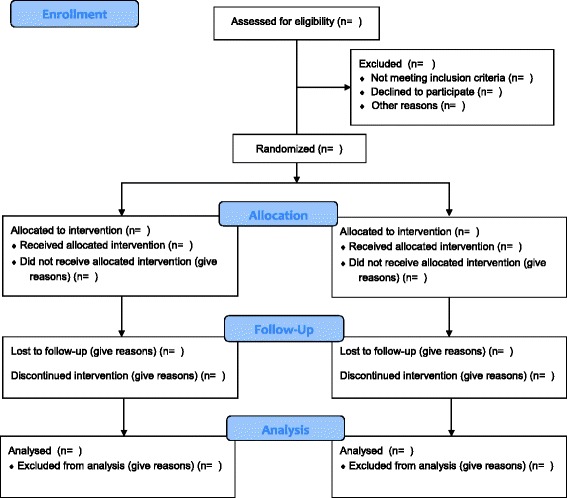
Consolidated Standards of Reporting Trials (CONSORT) flow diagram. Showing flow of participants through the trial

CIRCLE is a rater-blind, randomised controlled trial with two arms (Fig. 1). The experimental group will receive a 12-week CM intervention, as well as a manualised psychoeducational intervention delivered by clinical staff, which represents an Optimised version of Treatment as Usual (OTAU) that is offered by EIPs in the management of cannabis misuse. The control group will receive OTAU only. Assessments will be performed at the time of consent, 12 weeks following consent (at the end of the intervention period), and at 18 months following consent. The primary outcome is time to relapse, operationalised as admission to an acute mental health service.

### Recruitment

Recruitment to the trial will be in EIP services throughout the Midlands and the South East of England. Participants will be on the caseload of an EIP service and aged 18–36. Other inclusion/exclusion criteria are listed below.

### Group allocation

Following pretrial assessments, consenting clients will be randomised to group, stratified on severity of cannabis use (one to three uses per week, more than three uses per week). A remote, impartial randomisation service will manage the allocation to groups coordinated by the PRIMENT Clinical Trials Unit based at University College London (UCL).

### Trial assessors

Outcome assessments will be performed by trial research staff. Primary outcome assessors will be blinded to randomised allocation. Secondary outcome assessors will be blinded at the 18-month assessment interview. Research staff will be trained in the use of all measures by members of the CIRCLE team. Joint ratings with one another and with senior members of the team supervising them will be used to establish reliability.

### Interventions

The OTAU package will provide the context in which we will test the impact of a CM intervention. The CM intervention will involve the offer of voucher rewards for cannabis-free urine samples over a 12-week period to recent cannabis users with first-episode psychosis (defined below). We will first describe OTAU, delivered to both experimental and control groups, and then the CM intervention to be received by the experimental group only.

### Optimised Treatment as Usual (Psychoeducational package)

To be confident that we are measuring the effects of CM, a psychoeducational intervention will be delivered to both experimental and control groups. Guidelines on EIP care recommend that psychoeducational interventions for cannabis should be an important component of routine care, but consultations with EIP managers and staff suggest that the extent to which this is realised in practice is very variable between services and individual clinical staff. Our aim is thus to create a standardised version to be delivered by staff working in EIPs recruiting to the trial. A training manual for delivering the package, and supporting materials will be provided by trial staff to clinicians delivering OTAU. The intervention is designed to be sufficiently highly structured for staff without high-level clinical qualifications, such as support workers or assistant psychologists, to be able to deliver it competently following brief training.

OTAU has been designed to be an individually tailored psychoeducational approach to cannabis use for generic EIP clinicians, which applies general psychoeducational approaches used in first-episode psychosis [26]. It draws on the psychoeducational package offered in the control arm of a previous Melbourne pilot study of psychological intervention for cannabis use, the Cannabis and Psychosis (CAP) trial [27]. The package is comprised of six modules to be delivered via a standard PC. Full delivery of all six modules is typically achieved over approximately 3 h, normally offered over six regularly programmed sessions of 30 min duration. The package includes a pdf, video material, short quizzes, audio files, and further information and written records of the modules for the service user to keep. The material will remain focused on providing information in accordance with psychoeducation procedures, and will not act as a psychological intervention. The clinician’s main aim is harm minimisation, with an acknowledgement that in a young person with psychosis, cannabis abstinence may be required to ensure that no harm is done. The content is based on MI principles, relapse prevention, and harm-reduction strategies.

Psychoeducational materials including video, written, and web-based materials will present current information on the potential advantages and disadvantages of cannabis use and of cannabis abstinence. To help the participant to make an informed decision about continued use, the EIP staff will discuss the positives and negatives of cannabis use by exploring its impact on seven areas: family, finance, activity/engagement in work or education, mental health, physical health, legality, and social groups/friendships. Finally, staff will discuss setting goals regarding the young person’s future use of cannabis in the context of harm minimisation, as well as strategies for achieving their goals and avoiding relapse into patterns of cannabis use that compromise those goals.

### Contingency management

The CM intervention offers financial incentives contingent on urinalysis results indicating cannabis abstinence. The intervention voucher schedule and rules are adapted from Budney et al. [28, 29], which offered a voucher-based CM intervention for treatment of cannabis dependence in the general population. The intervention comprises 12 once-weekly urinalysis sessions and will be delivered by EIP clinicians. At each session, the participant will be required to provide a urine sample. A temperature strip on the side of the specimen cup will allow the EIP staff to check whether the sample has been tampered with. In week 1 of the intervention, details of the intervention will be explained to the participant, and they will be asked to sign an ‘abstinence contract’ indicating that they understand and accept its rules, and agree to abide by the test results. In the first week, participants will receive a £5 voucher for attending and providing a urine specimen independent of the drug test results, which provides a ‘baseline’ result. From week 2 to week 12, participants will receive vouchers, increasing by £5 every 2 weeks, contingent upon producing negative specimens. Vouchers will be for a local supermarket. Participants who abstain from cannabis use for the full duration of the intervention will earn £240.

Urinalysis will be performed using a small benchtop analyser capable of providing rapid test results of drug misuse urinary concentration (Kaiwood CHR-110). To perform the analysis, the EIP staff member pipettes a fixed amount of urine into a buffer solution tube to give a 7:1 serial dilution. This allows a standard 50-ng/ml marijuana test cassette placed in the analyser to provide a urinary cannabis concentration reading between 0 and 350 ng/ml. Guidelines will be provided to the EIP staff to allow interpretation of the test results, whereby a sufficient drop in urinary tetrahydrocannabinol (THC) concentration will be taken as indicative of abstinence since the previous urinalysis session. These guidelines are based on published data regarding the urinary half-life of cannabis [30].

Participants are able to prearrange missing scheduled sessions (‘holiday week’) and still receive the reward for that week if they have a valid commitment that prevents them from attending. They can do this on a maximum of two occasions for 1 week only each time. They will still be expected to show evidence of abstinence at the following session to receive a reward for the holiday week. If the participant misses the following week or provides a positive sample, no financial incentive will be received for the holiday week. Holiday weeks need to be arranged with the staff member performing the intervention no later than at the time of the previous scheduled appointment. The intervention will be suspended for a maximum of 1 month if a participant relapses or otherwise loses mental capacity. If capacity is not regained in 1 month, the intervention will not continue. If a participant fails to attend on multiple consecutive weeks, or if contact is lost with the participant entirely, each missed week will be counted as a failure to attend.

Failure to attend intervention sessions, specimens suggesting cannabis use, or failure to submit a scheduled specimen will reset the value of vouchers back to the initial £5.00. If the participant attends the next week and provides a negative sample, they will be rewarded with £10. In the subsequent week, if the participant provides a second negative sample voucher values will continue from the highest previous level of reward.

### Selection and training of staff

Staff in the EIP services will deliver the CM and OTAU interventions. Training will be delivered to all staff delivering the interventions by members of the research team over a period of half a day on average.

### Inclusion/exclusion criteria

#### Inclusion criteria

The cohort will be EIP service users who have recently abused cannabis. Recent cannabis use is operationalised as having used cannabis at least once during 12 of the previous 24 weeks. Additional eligibility criteria include (1) being aged 18–36, (2) having stable accommodation (i.e. not street homeless or roofless), (3) speaking enough English to be able to understand fully and answer the assessment instruments, and (4) being able to give informed consent. EIP teams have been set up across England following the 2000 NHS Plan [31]. Standard criteria for EIP include developing symptoms of psychotic illness for the first time, with positive psychotic symptoms persisting for at least a week and accompanied by evidence of significant risk and/or functional decline. Service users are typically discharged after 3 years on the caseload of an EIP team.

#### Exclusion criteria

Exclusion criteria include (1) those who fail EIP service inclusion criteria, (2) those currently engaged in treatment for cannabis use with another agency, (3) those currently compulsorily detained in hospital or prison, or (4) those on probation or Community Treatment Order requiring drug testing for cannabis.

### Obtaining informed consent

In the first instance, a member of the EIP staff will obtain agreement from potential participants to being contacted by a member of the CIRCLE research team. The researcher will then meet with the service user to provide a participant information sheet, written in plain English, which will explain all aspects of the study. They will also explain all benefits of the study and known risks. The service user will be given at least 48 h to consider participation prior to consent being taken.

### Ethical approval

Ethical approval for the trial was received on 16 March 2012 from the London – South East NRES Committee (REC reference 11/LO/1939). Written informed consent will be obtained from all participants in the trial. The original consent forms will be stored at the author’s institutions (UCL, KCL, University of Sussex, and Warwick University), and a copy will be kept in the patients’ clinical notes.

### Assessment interviews

Participants will be given three assessment interviews: at the time of consent, at 12 weeks following consent, and at 18 months following consent, a time at which a significant proportion of young persons with psychosis will relapse if they are going to do so [32, 33]. All participants will be given a £20 voucher for their time, and at the follow-up assessment all participants will be given an extra £10 for the provision of a urine sample. At 18 months, the primary outcome and some secondary outcome data will also be collected from electronic patient records.

### Outcome measures

**Fig. 2:**
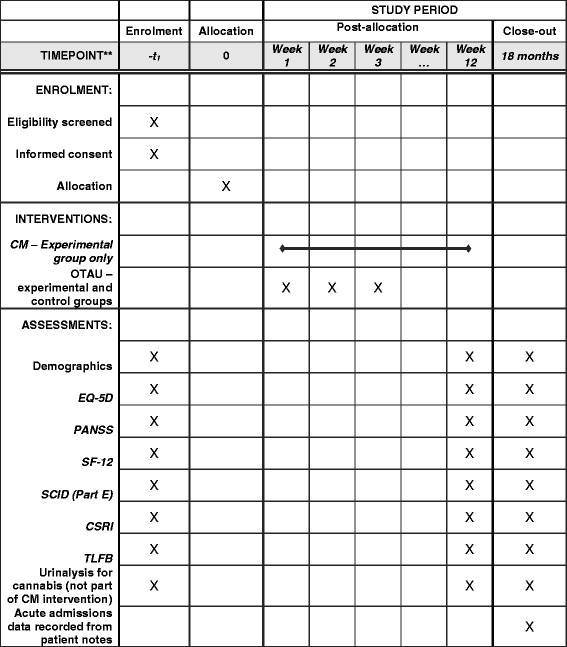
Standard Protocol Items: Recommendations for Interventional Trials (SPIRIT) figure. Overview of the schedule of events

At all assessment interviews the following measures will be performed in addition to the collection of standard demographic information (Fig. 2):

- Cannabis use
  - ○ The Time Line Follow Back (TLFB) [34] will be used to record self-reported cannabis use over the last 6 months. The TLFB is a retrospective calendar-based measure of daily substance use, with good test-retest reliability demonstrated for cannabis [35]. This will be used to establish eligibility in terms of cannabis use and extent of recent use
  - ○ Structured Clinical Interview for the *Diagnostic and Statistical Manual of Mental Disorders, version 4* (DSM-IV) (SCID) part E will be used to assess history of alcohol and substance misuse disorders
  - ○ Specimens for urinalysis will be obtained with the threshold set at a level for detecting cannabis use in the previous 28 days (i.e. 50 ng/ml cannabis metabolites)
- Psychotic symptoms
  - ○ The positive and negative subscales of the Positive and Negative Syndrome Scale (PANSS) [36]. The PANSS is a well-established measure of psychotic symptoms, with strong psychometric properties in terms of validity, reliability, and sensitivity [37]
- Service use and health economic analysis
  - ○ Client Service Receipt Inventory (CSRI), developed originally by Beecham and Knapp [38] will be used to record clinical service use, medication use, receipt of state welfare, and use of other state-funded services including criminal justice services
  - ○ The 12-Item Short Form Survey (SF-12) [39] and the EuroQoL, 5 dimensions (EQ-5D) questionnaire [40] are widely used measures of health status with good psychometric properties [41, 42], which will be used to derive quality-adjusted life years’ (QALYs) data

### Primary outcome

The primary outcome will be time to relapse. Admission to a psychiatric hospital, crisis resolution team or crisis house, or other acute mental health service will be used as a marker of relapse. The primary outcome will be assessed at 18-month follow-up based on electronic patient records. Dates of admission will be recorded and participants will be followed until they have relapsed, are lost to follow-up, or until the end of the 18-month study period.

### Secondary outcomes

Secondary outcomes will be collected mainly through assessment interview, and will include:

- Cannabis use, including self-reported use and urinalysis results at follow-up
- Positive symptom severity (Positive and Negative Syndrome Scale [36])
- Social functioning, based on self-reports of engagement in work or study
- Number of days cannabis use in the previous 12 weeks (for 12-week follow-up) or 6 months (for 18-month follow-up)
- Number of admissions over 18 months’ follow-up
- QALYs (SF-12 and EQ-5D) [43] and service use (CSRI) will be used in the cost-effectiveness analyses, as described in the analysis section below. Service utilisation data will be augmented where possible from participants’ medical records at 18 months

### Proposed sample size

Our sample size for the main trial is based on data suggesting a usual relapse rate of around 50 % over the study timeframe in cannabis users [3, 5]. A 15 % decrease in this relapse rate due to the intervention is clinically beneficial. Using a power of 90 % and a significance level of 5 %, a total sample size of 460 subjects will be required. This sample size is based on an analysis of time to relapse and will allow us to detect a 37 % decrease in the hazard of relapse (hazard ratio of 0.63) in the intervention group using a Cox proportional hazards model. This sample size has been calculated using Stata version 11 [44]. The sample size is inflated by a factor of 1.06; assuming that each person delivering the intervention sees an average of four service user participants in the trial, and an intraclass correlation coefficient of 0.02 for clinician clustering, this gives a total sample size of 488. Finally, the sample size is inflated by 10 % to account for attrition for the primary outcome, giving a total sample size of 544.

### Statistical analysis

A detailed analysis plan will be written and signed off by the Data Monitoring and Ethics Committee for the trial before the analysis commences. Initial analyses will look at summary statistics for all variables, both overall and by randomised group. Summary statistics for continuous variables will be mean, median, standard deviation (SD), lower quartile, upper quartile, and minimum and maximum and will be reported appropriately according to distribution. Summary statistics for categorical variables will be frequency and percentage within each category.

Summary statistics of baseline variables by whether a participant has dropped out of the study will also be examined to determine whether the dropouts had similar characteristics to those who remained in the study at baseline.

After checking the assumptions of proportional hazards, Cox proportional hazards modelling to compare the intervention and control groups, adjusting for severity of cannabis use at baseline (dichotomous – one to three times a week versus four or more times a week), will be carried out for the primary outcome. Robust standard errors will be used to account for clustering by care coordinator. If the assumption of proportional hazards is not fulfilled, alternative modelling strategies will be employed. The secondary outcomes will be analysed using appropriate regression models, separately for data collected at 12 weeks and 18 months. Estimates and 95 % confidence intervals will be presented for the secondary outcomes. In a supportive analysis, all analyses will be adjusted for an indicator as to whether or not the participant was in the pilot trial and for potential baseline predictors of missingness related to outcome. All analyses will be carried out on an intention-to-treat basis using all available data.

### Missing data

It is expected that there will be few missing data for the primary outcome as data for this will be extracted from the participants’ medical records. There is likely to be more missing data for the secondary outcomes as the majority require the participant to be interviewed to complete the measures. For both the primary and secondary outcomes we will check the extent and patterns of missing data and identify predictors of missingness. Multiple imputation or adjustment for potential predictors of missingness related to the outcome will be performed if appropriate.

### Economic evaluation

For the health economic analysis, intervention costs will be calculated using available data on staff costs, incentives, on-costs, other overheads, and activity levels. These will be added to the costs of other health and social care services derived from the Client Service Receipt Inventory and records combined with nationally applicable unit costs (e.g. Curtis [45]). Cost comparisons at 3 and 18 months will be made using regression models, with bootstrap methods used to generate confidence intervals around the cost differences. Cost-effectiveness from an NHS perspective at 3 and 18 months will use three outcome measures: number of cannabis-negative urine samples, days of reported cannabis abstinence and QALYs (derived from the EQ-5D with SF-12 QALYs used in secondary analyses)). If, for any of these the intervention has higher costs and better outcomes than usual treatment, then cost-effectiveness will be expressed in the form of incremental cost-effectiveness ratios, estimated by dividing the incremental costs by the incremental benefits of the intervention. Uncertainty around cost-effectiveness estimates will be explored using cost-effectiveness planes (through generating a large number of cost-outcome combinations using bootstrap methods) and cost-effectiveness acceptability curves (showing the probability of the intervention being cost-effective at various levels of willingness to pay for health benefits). The range of values for QALYs will be £0 to £100,000 so as to include the threshold used by NICE. The values for the other measures will be chosen so that the points at which one arm has 50 %, 60 %, 70 %, 80 %, and 90 % of being the most cost-effective can be observed. It will then be a value judgement as to whether these values are acceptable. Cost-effectiveness will be investigated regardless of clinical outcome.

## Discussion

The present study is a rater-blinded RCT investigating the clinical and cost-effectiveness of CM for reducing cannabis use in EIP patients with a history of recent cannabis use. Cannabis is a significant issue in this population. Rates of cannabis use among people with first-episode psychosis are high, resulting in poorer clinical, social, and functional outcomes, and greater clinical service use. However, there is little evidence for any effective interventions for comorbid substance misuse in established psychosis. If CM is found to be clinically and economically beneficial, it will offer strong support for using such interventions to reduce cannabis use among EIP service users.

### Strengths and limitations

With a recruitment target of 544 participants, CIRCLE is one of the largest trials of CM worldwide. To the best of our knowledge it is also the first RCT of CM specifically for cannabis use in psychosis, although there are a number of related studies. Bellack et al. [23] trialled CM for substance use in psychosis, including cannabis, cocaine, or heroin, which required participants to test negative from the first session. Sigmon et al. [24, 25] performed two small feasibility studies of CM for cannabis use in psychosis, but used within-subject designs rather than a RCT design. Participants are followed over a relatively long period of 18 months in accordance with NICE recommendations for future research for psychosocial interventions for substance misuse in psychosis [46]. The rationale for targeting EIP patients in particular is that there is also good reason to believe that motivation to change patterns of cannabis and other substance use is high in this cohort [15]. Secondly, EIP is a form of secondary preventative care [47], with the aim of preventing or attenuating the risk of relapse to improve long-term prognosis. Given the substantial evidence base linking cannabis use to higher rates of relapse, reducing cannabis use in EIP services is consistent with EIP aims.

One potential concern regarding the use of CM interventions in publicly funded health services is its acceptability to the public, clinicians, and service users. A mixed-method substudy to the main trial to explore this topic is planned. However, there is already some evidence that public opinion is in favour of CM for treatment adherence in severe mental illness [48]. Potential concerns about the use of financial incentives were also carefully considered in the design of the CIRCLE intervention. The design is based on Budney et al. [28, 29] and feedback on it was sought from service users, pilot study participants, carers, and clinical teams before and after the pilot study through focus groups and one-to-one interviews.

One technical issue for CM for cannabis use is the relatively long effective half-life of cannabis. Conventional marijuana urinalysis tests could not be used for the CM, as a positive urine result may be related to cannabis use that had taken place more than 1 week previously. Use of such tests would delay the initiation of treatment by up to 4 weeks to allow a participant’s urinary THC level to fall below 50 ng/ml. To address this, CIRCLE uses desktop analysers capable of providing a urinary THC concentration reading. As discussed, a reduction in urinary THC in line with trial guidelines will be taken as evidence of abstinence. However, it is possible that a reduction in urinary THC over a 1-week period could occur due to a reduction in cannabis use rather than abstinence. As such, it is possible that participants can receive the voucher reward while still using in the short term. However, medium and long-term trends, detectable over two or three sessions, will clearly indicate abstinence rather than reduction of cannabis use as urinary THC will not continue to decline or fall to below 50 ng/ml. Participants will be informed that they will need to abstain fully throughout the intervention period to receive all the voucher rewards.

### Trial status

The pilot phase of CIRCLE began on 1 January 2012 and ended on 28 February 2013. Approval to proceed to the full trial was received in April 2013 and recruitment to the main trial is currently ongoing. The end date for CIRCLE is 31 October 2017.

## Abbreviations

CBT: Cognitive behavioural therapy
CM: Contingency management
EIP: Early Intervention in Psychosis
MI: Motivational interviewing
NICE: National Institute for Health and Care Excellence
OTAU: Optimised Treatment as Usual
RCT: Randomised controlled trial
